# Ultra-low-loss tunable piezoelectric-actuated metasurfaces achieving 360° or 180° dynamic phase shift at millimeter-waves

**DOI:** 10.1038/s41598-020-72874-y

**Published:** 2020-09-24

**Authors:** Evangelos Vassos, James Churm, Alexandros Feresidis

**Affiliations:** Department of Electrical, Electronic and Systems Engineering, University of Birmingham, Birmingham, B15 2SA UK

**Keywords:** Engineering, Electrical and electronic engineering

## Abstract

Phase shifting metasurfaces typically consist of an ordered metallic geometry that is patterned onto a dielectric substrate and incorporate active devices or materials that enable dynamic tuning. Existing methods at mm-wave and submillimeter bands typically suffer from high losses, which are predominantly produced by the inherent limitations of the tuning elements or materials. This report presents a new, ultra-low-loss and phase-tunable, reflection type metasurface design, which outperforms previously reported technologies in terms of phase shifting and loss. The proposed technique utilizes a variable air cavity, formed between a periodic array and a ground plane, which is controlled by means of a piezoelectric actuator. Two metasurface designs are presented and experimentally tested. Firstly, a square patch element metasurface that is capable of achieving a continuous 180° phase shift across a wide bandwidth, between 35 and 65 GHz. Also presented is a double-cross element metasurface that provides full 360° phase control between 57 and 62 GHz. The variable air cavity is controlled by means of a piezoelectric actuator that supports and varies the height of a ground plane, providing highly accurate, millisecond, displacement. Unlike conventional tuning methods, the tuning mechanism, in this case the moving ground plane, introduces no additional sources of loss and enables an average loss performance of 1 dB. Full-wave simulations are presented and experimentally validated with measurements of both metasurface prototypes. The proposed approach is scalable from microwave up to THz frequencies, due to the electro-mechanical and low loss nature of the tuning.

## Introduction

Metasurfaces act as planar structures that can manipulate an incident electromagnetic wave in unconventional ways that are not possible with normal materials^1–3^. Typically, metasurfaces are made up of a bi-dimensional periodic lattice of subwavelength metallic elements on a dielectric substrate. Such structures can be engineered to act as artificial impedance surfaces that produce a controlled reflection response to an incident wave. Controlling the reflection phase of the incident wave is essential in many radio frequency, microwave and THz applications, including: propagating mode to surface mode conversion^4^, anomalous reflection^5^, reflectarray antennas^6–12^, absorbers^13–15^, high impedance surface based antennas^16^ , transmitarrays^17–19^ and anisotropic polarization converters^20^.

The geometry of an artificial impedance surface, along with its constituent materials affect the periodic array resonance and consequently, the reflection phase. In typical implementations of a reflection phase-shifting surface, the array is printed on a grounded substrate and tuning of the reflection phase is achieved by the incorporation of an active material or device. The active elements add an extra, and dynamically variable, degree of freedom in any of the metasurface parameters to produce a tunable reflected response^21,22^. Various tunable components have been previously proposed, including: ferroelectric substrates^23^, liquid crystals^24,25^, phase change materials such as vanadium dioxide^26^, graphene^27–29^, diodes^30–33^, microelectromechanical systems (MEMS)^34–38^ and more recently piezoelectric actuators (PEAs)^39^. The benefits of any given technology depend very much on application specific factors such as: frequency of operation, loss tolerance, switching/tuning speed, ease of integration, form factor, cost etc.

Wide operating bandwidths, a full 180° or 360° phase control, and minimum losses are of particular importance for tunable metasurfaces. However, the majority of thus far reported tunable components or materials have limitations at mm-wave frequencies, mainly in terms of high losses, slow tuning/switching speeds, or difficulty in achieving full 360° phase shifting. PEAs on the other hand, offer a new design approach that requires a different implementation when compared to other reported tunable phase shifter metasurfaces. A PEA enabled metasurface, comprising of a periodic metallic array suspended over an adjustable ground plane, has previously been reported to produce a very narrowband phase shifting response due to the limited displacement provided by the actuator and the particular design of the unit cell^39^. Phase shift was also limited due to the single element nature of the reported unit cells^40^.

**Figure 1:**
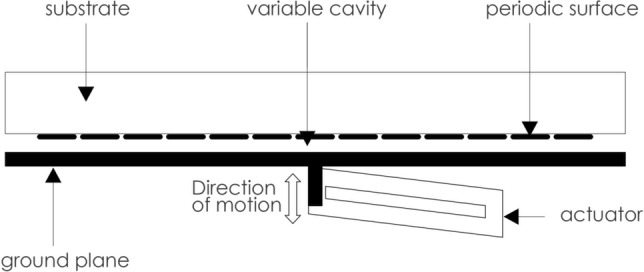
A schematic of a piezoelectric actuator controlled phase shifter. The variable cavity formed between the periodic metallic elements and the ground plane is controlled by the application of voltage to the piezoelectric actuator. This results in the ground plane moving along the axis of motion.

In this paper, we present a new class of continuously tunable metasurfaces, enabled by a flexure amplified linear piezoelectric actuator (PEA), exhibiting unprecedented low-loss phase shifting performance at mm-waves. A periodic array of subwavelength metallic elements is fixed above a ground plane so that an air cavity separates them. The ground plane is mounted onto the PEA such that its position can be moved either closer to or further away from the periodic array. The displacement of the ground plane produces a variation in the air cavity thickness, which can be dynamically controlled by applying a voltage to the PEA. The variation of the cavity thickness changes the reflection response of the metasurface under plane wave incidence, which enables control of the reflected phase. A schematic of the proposed technique can be seen in Fig. 1.

We present two low-loss phase shifting metasurfaces: the first is a very wide band tunable metasurface achieving a continuous 180° phase shift between 35 and 65 GHz; the second is a full 360° phase control tunable metasurface between 57 and 62 GHz. The proposed designs produce ultra-low-loss and continuous phase shifting due to the fact that the tuning is electro-mechanical in nature, and the element used to provide the displacement (the PEA) is mounted below a shielding ground plane and therefore does not become part of the mm-wave circuit that it is affecting. The tuning is controlled by the continuous and accurate displacement (of the order of micrometer resolution) provided by the PEA under an applied analog DC bias voltage, which results in continuous tuning which is beneficial in many applications.

## Results

### Wide bandwidth metasurface

**Figure 2:**
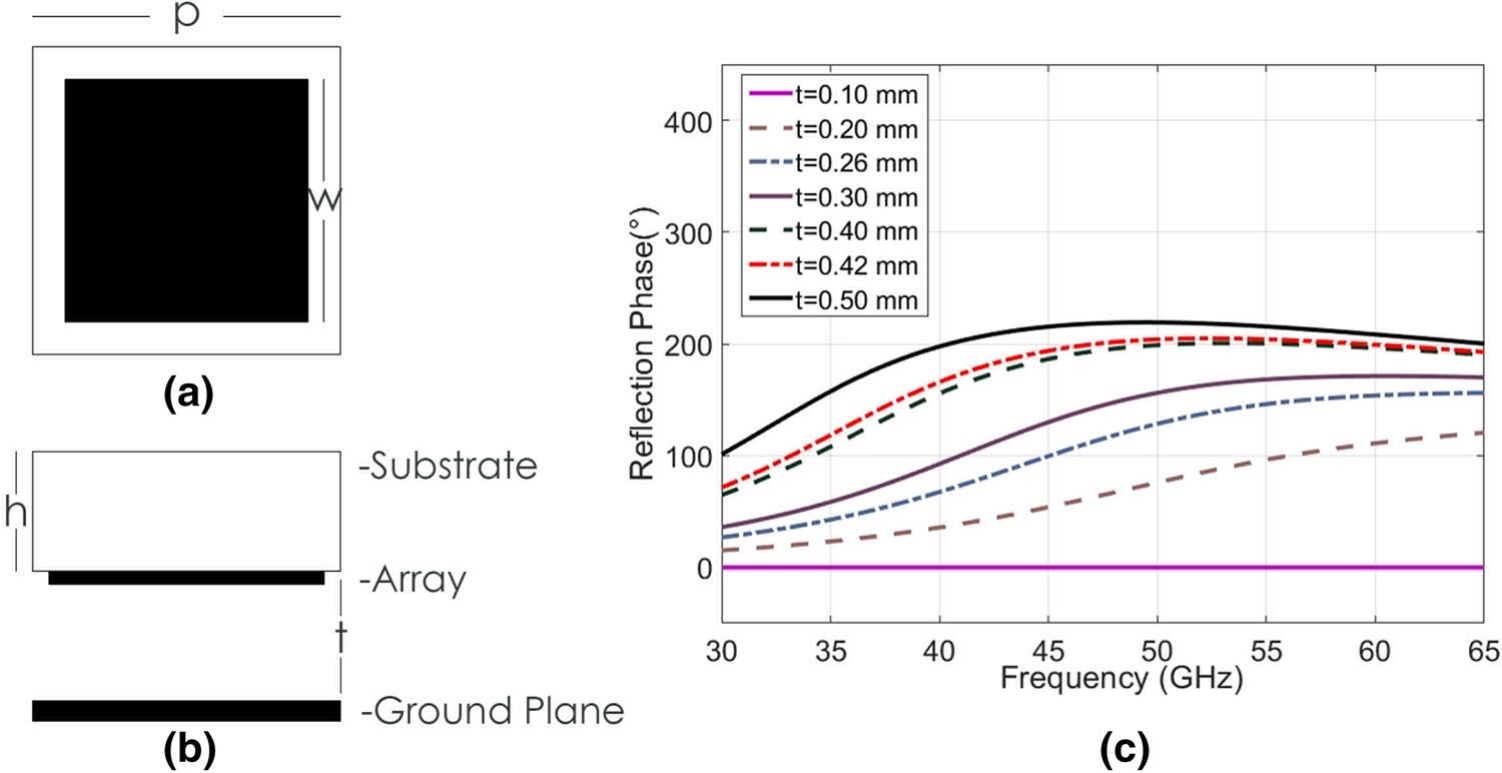
The proposed wide-bandwidth unit cell. (**a**) Schematic of the metallic surface of the proposed single element unit cell, where w = 1.22 mm and p = 1.36 mm. (**b**) Schematic of the side view of the proposed single element unit cell, where h 0.78 mm and t is variable between 0.10 and 0.50 mm. (**c**) Simulation results of reflection phase difference of the proposed single element unit cell by varying the parameter t. Results are presented using the reflected phase corresponding to 0.10 mm cavity height as reference.

The first of the proposed metasurfaces is comprised of a periodic array of square metallic patch elements, as seen in Fig. 2a,b where the periodicity, *p*, is 1.36 mm and the patch width, *w*, is 1.22 mm. The dielectric thickness is 0.78 mm and the dielectric constant is 2.2. Initial simulations were conducted with a single unit cell that interacts with a normally incident plane wave, using periodic boundaries to approximate a large periodic array of elements. These simulations suggested that a reflected phase shift of over 180° could be expected between 35 and 65 GHz when *t*, the air cavity height (Fig. 2b), was varied between 0.10 and 0.50 mm.

**Figure 3:**
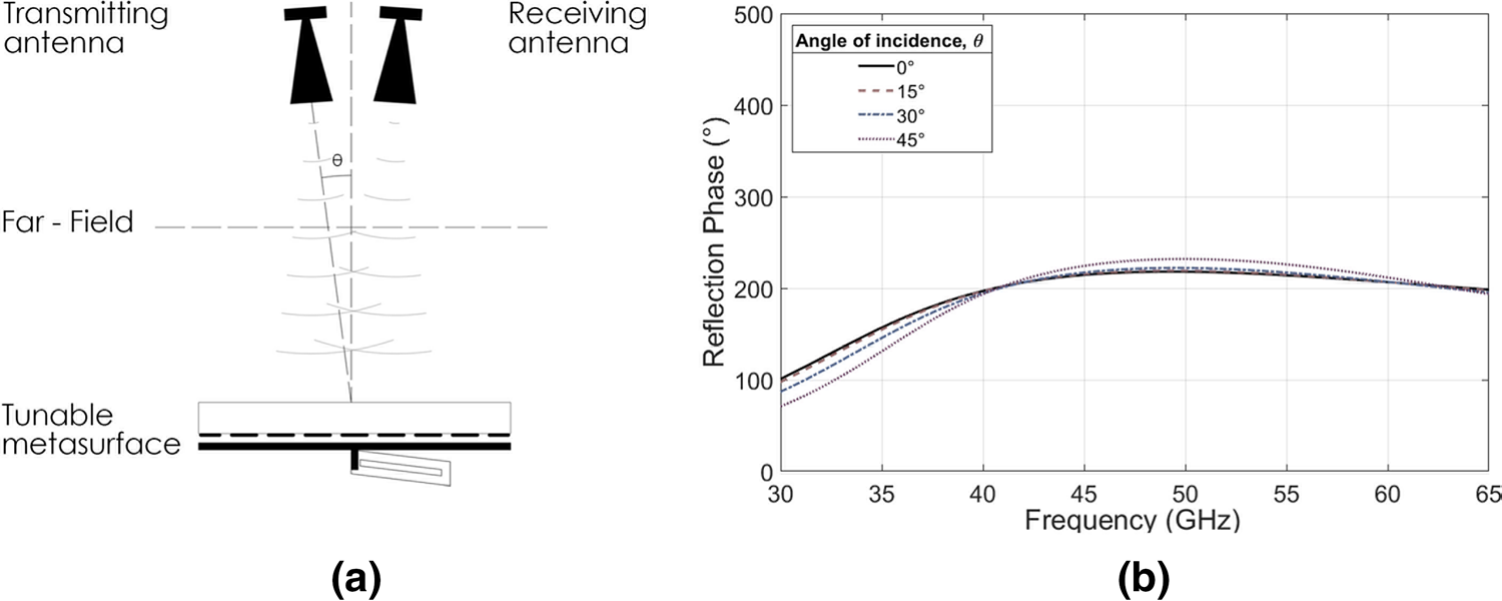
Effects of angle of incidence. (**a**) Schematic of the scenario setup. (**b**) Simulation results of the maximum reflection phase difference of the proposed single element unit cell by varying the angle of incidence θ from 0° to 45°.

As the unit cell element in this design is doubly symmetric, the phase shifting performance remains consistent in both orthogonal polarizations. The dimensions *p* and *w* were optimized to their presented values for bandwidth, phase shift and low-loss operation. This results in a maximum phase shift of 219° at 50 GHz, and a continuous > 180° phase shift between 35 and 65 GHz, which is tuned with a cavity adjustment of up to 0.40 mm (Fig. 2c). The angular stability for θ as in Fig. 3a, for TE incidence shows little deviation in maximum achievable phase shift from 0° to 45°, as in Fig. 3b. The results for TM incidence are similar (omitted here for brevity) and both modes exhibit losses less than 1 dB across the functional bandwidth.

The fabricated metasurface consists of a periodic array of these unit cells, patterned onto a dielectric and suspended above a ground plane, creating the air cavity with height, *t*. The substrate is not included in the cavity in order to minimize reflection losses and maximize the impact of cavity height variation. The proposed tuning technique utilizes a PEA that is mounted on the underside of the ground plane and provides the cavity height variation which results in a continuous low-loss phase shifting. This implementation was realized on Taconic TLY-5 substrate of 0.78 mm thickness in an array 100 mm × 100 mm, which represents approximately 10 λ × 10 λ at the lowest operational frequency.

For experimental assessment, the periodic array was fixed in position, and the ground plane mounted to the PEA, which was held at 0 V. This produced between a 0 and 0.1 mm separation between the periodic surface and the ground plane. The uncertain uniformity here is due to the lack of flatness of the substrate producing uneven separation. When applying a DC voltage bias of 120 V to the PEA, the separation increased to around 0.5 mm.

**Figure 4:**
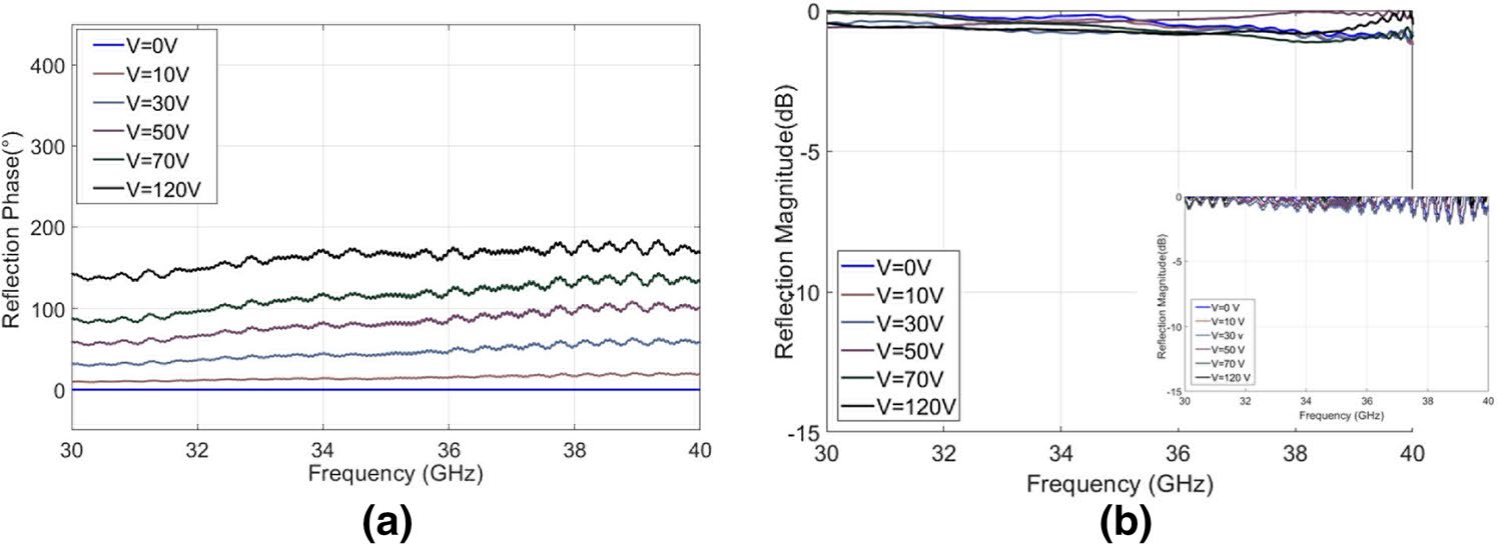
Wide bandwidth metasurface (30–40 GHz). (a) Reflection phase shift with respect to 0 V (approx. 0.10 mm cavity height) state. (b) Average reflection magnitude for different applied voltages (raw data shown in insert).

The design of the PEA is such that relative flatness is maintained and no gradient is imparted to the ground plane during actuation. Taking care with relative alignment of the two surfaces, the voltage was increased in order to move the ground plane away from the periodic surface. A reflection measurement was then taken, at various applied voltages. Figure 4a shows the measured reflection phase of the different voltage states, with 0 V results as reference. Figure 4b shows the measured losses, normalized with respect to the bare metal ground plane without the periodic surface at the respective displacement values.

**Figure 5:**
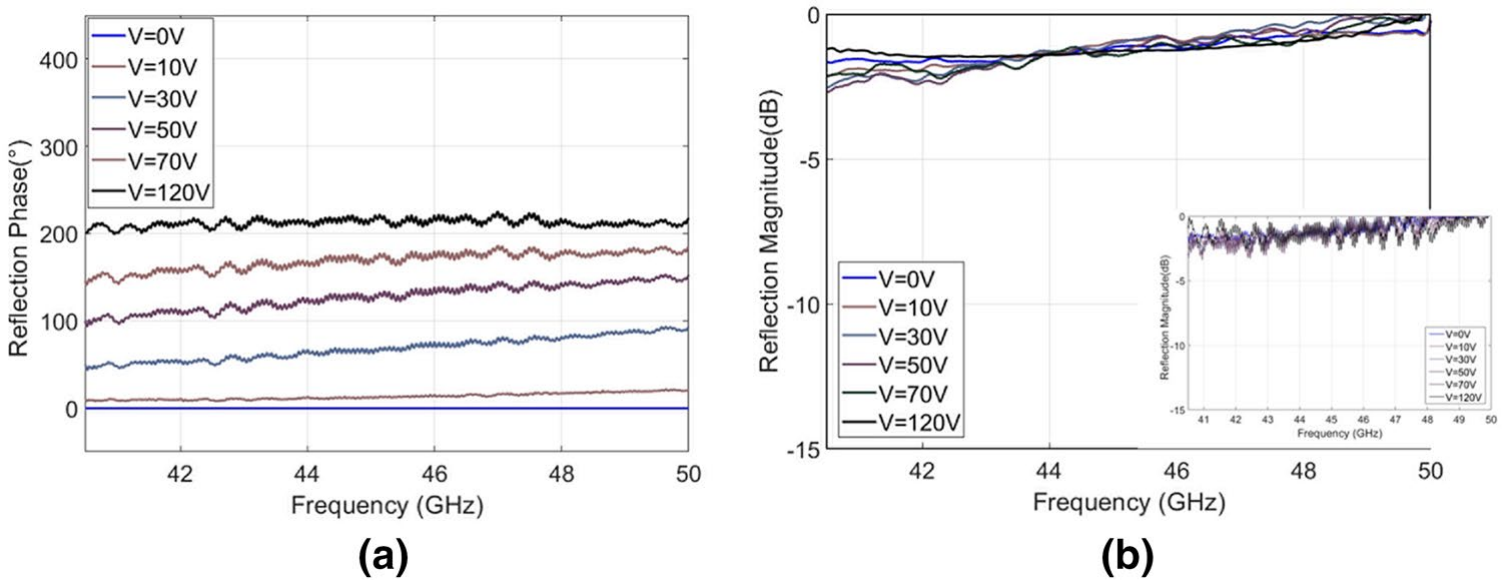
Wide bandwidth metasurface (40.5–50 GHz). (**a**) Reflection phase shift with respect to 0 V (approx. 0.10 mm cavity height) state. (**b**) Average reflection magnitude for different applied voltages (raw data shown in insert).

**Figure 6:**
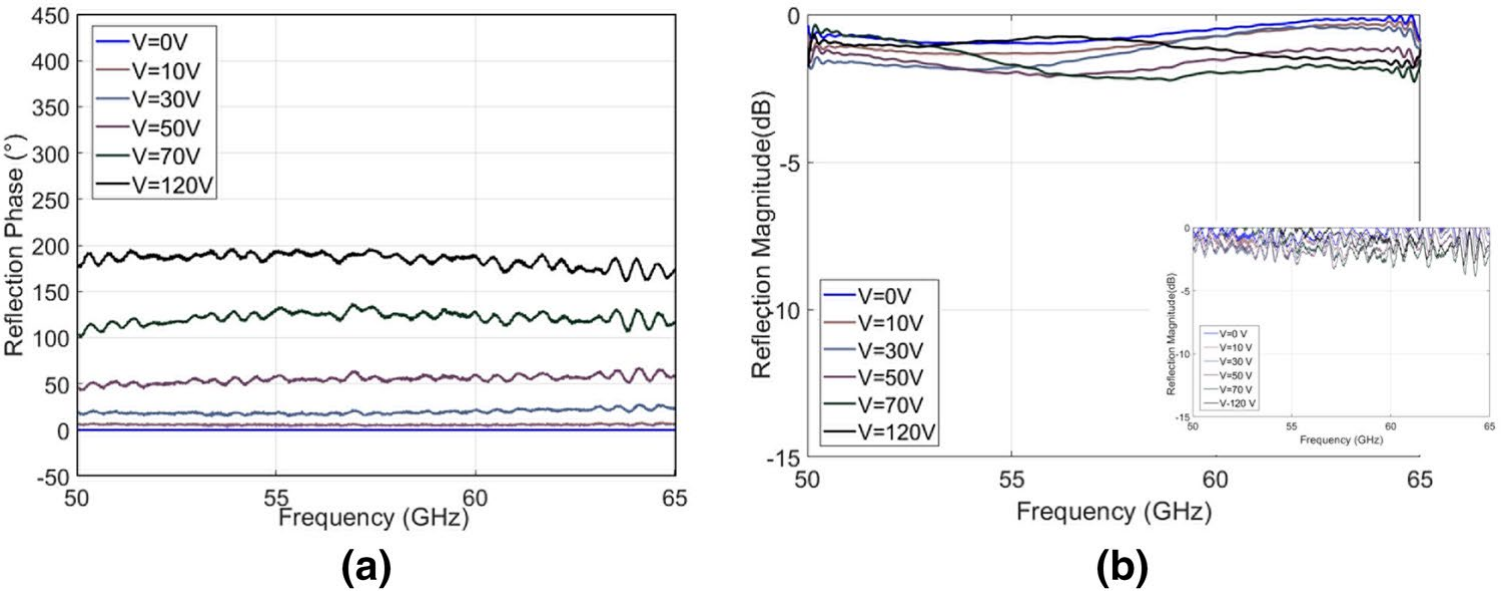
Wide bandwidth metasurface (50–65 GHz). (**a**) Reflection phase shift with respect to 0 V (approx. 0.10 mm cavity height) state. (**b**) Average reflection magnitude for different applied voltages (raw data shown in insert).

Due to the broadband nature of the metasurface performance, it was necessary to split the measurement over several antenna ranges. Two more sets of results were taken, one between 40.5 and 50 GHz, which can be seen in Fig. 5 and the other between 50 and 65 GHz, which can be seen in Fig. 6.

The maximum measured reflection phase difference is 219° at 47.5 GHz. The bandwidth with a phase difference of > 180° was verified and is in agreement with the simulation results, i.e. from 35 to 65 GHz (60% fractional bandwidth). The reflection magnitudes for each band are presented in Figs. 4b, 5b, 6b and the measured losses are on average 1 dB (always remaining less than 2 dB) over the entire bandwidth. There is good agreement between the measured and simulation results, the small discrepancies and fluctuations that appear in the measured results are most likely due to the mechanical compliance of the dielectric. A sagging was observable with approximate magnitude of 0.1 mm from center to the edges. There was also a slight but observable lack of parallel alignment between ground and periodic surface.

### Full 360° control

**Figure 7:**
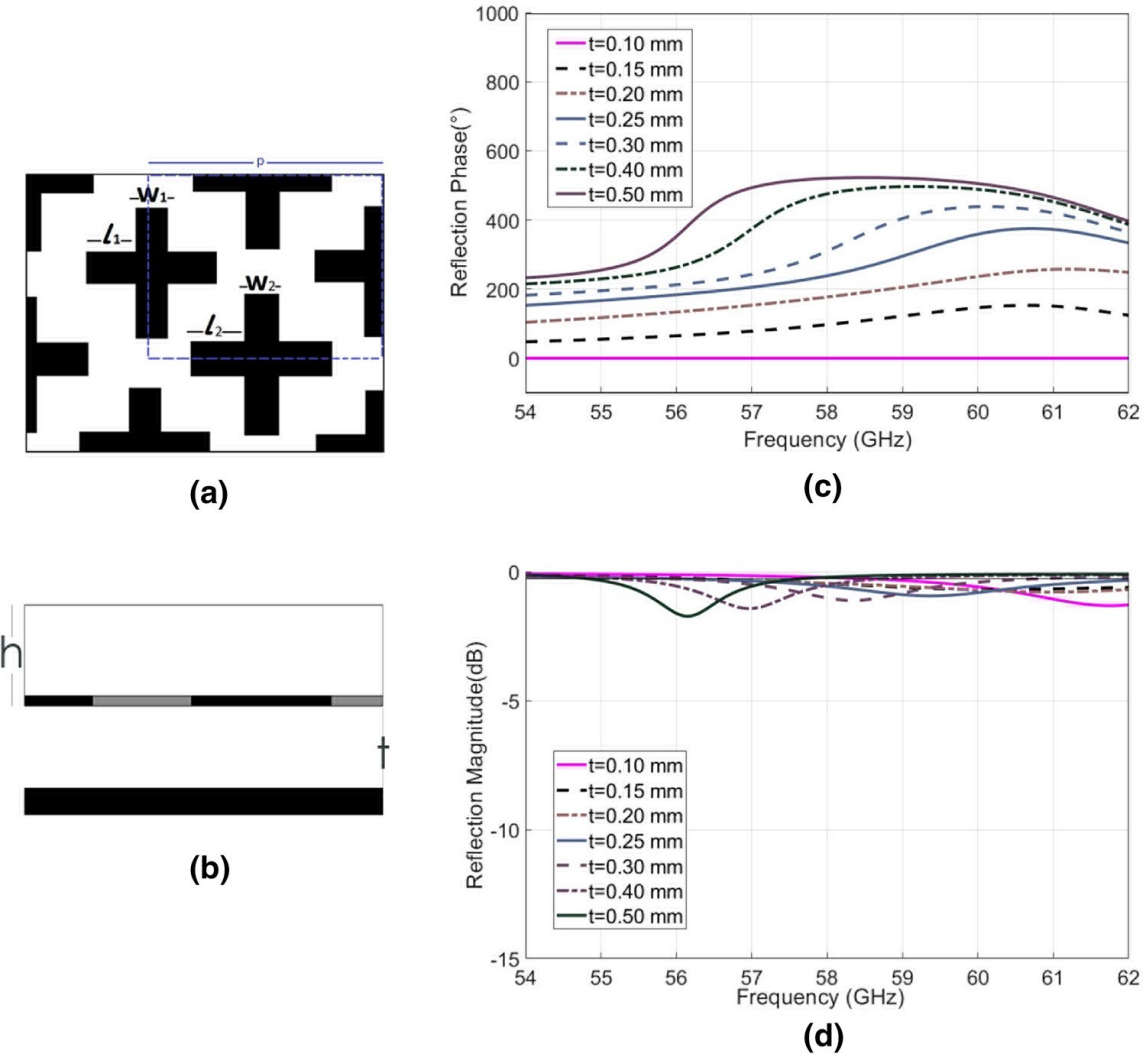
The proposed full phase control unit cell. (**a**) Part of the metasurface array with unit cell outlined by blue dashed line where w_1_ = w_2_ = 0.30 mm, l_1_ = 0.75 mm, l_2_ = 0.85 mm, p = 2.6 mm, h = 0.78 mm and t is variable between around 0 and 0.5 mm. (**b**) Side view of proposed unit cell with variable cavity separation, where t is variable between 0.10 mm and 0.50 mm, and h is 0.78 mm. (**c**) Reflection phase difference simulation results of the unit cell with varying t. (**d**) Reflection magnitude simulation results with varying t. Results are presented using the reflected phase corresponding to 0.10 mm cavity height as reference.

The second design is a novel phase-shifting reflection metasurface that produces full phase control of 360° in both TE and TM polarization. The unit cell in this model consists of two offset and dissimilar cross elements as outlined in blue in Fig. 7a. The two cross elements have the same arm width of *w*_1_ = *w*_2_ = 0.30 mm, but different arm lengths, *l*_1_ = 0.75 mm and *l*_2_ = 0.85 mm, with a total periodicity of 2.6 mm. The operation of the metasurface in all other ways is the same as the previous example, as it is suspended over a movable ground plane so that a variable cavity is produced beneath the metallic pattern (Fig. 7b).

The triangular lattice symmetry and slight difference between the two cross element dimensions cause a double resonance to be generated which enables a progressive reflection phase shift. The continuous 360° phase shifting performance is enabled by this progressive phase when combined with the displacement effects. It is possible to optimise this structure for further enhanced phase shifting with the same amount of displacement, however there is an inherent trade-off between loss performance and maximum achievable phase shift. An optimum design is presented here that yields full phase control with good loss performance. As with the previous example, the symmetric nature of the proposed surface means performance is maintained between TE and TM modes.

The double-cross unit cell was simulated using periodic boundary conditions. The gap between the periodic surface and the ground, *t*, was varied between 0.10 and 0.50 mm. The simulated results can be seen in Fig. 7c,d, and demonstrate an average loss of 0.5 dB (always remaining below 2 dB) across the operating bandwidth between 57 and 62 GHz, over which range a > 360° phase shift is achieved. The maximum phase shift is 530° at 58 GHz and is achieved by utilizing a 0.40 mm displacement.

**Figure 8:**
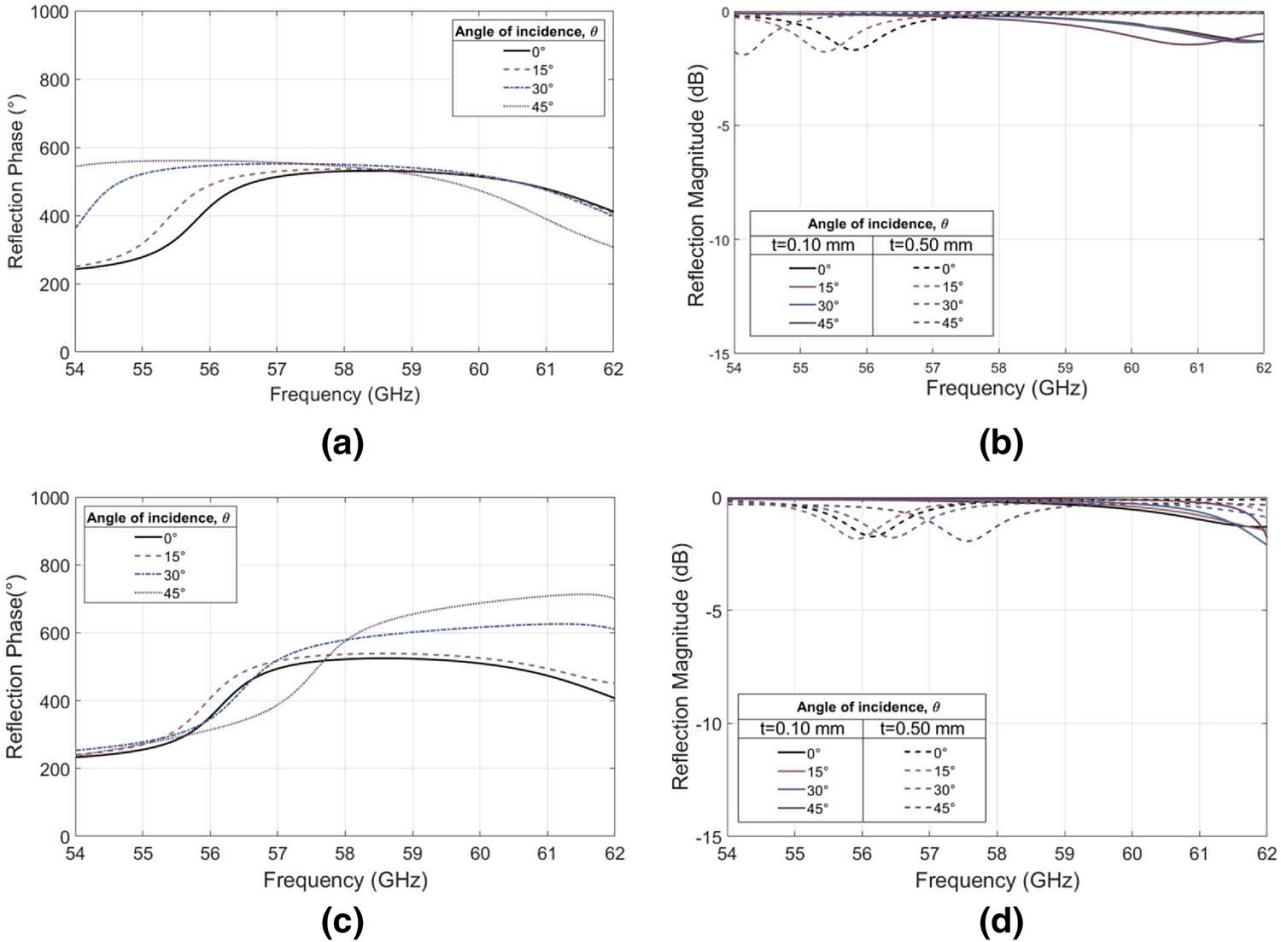
Angular stability study of the double-cross unit cell. (**a**) Simulated results of the maximum reflection phase difference by varying the angle of incidence θ from 0° to 45° for TE polarization. (**b**) Reflection magnitude simulation results with varying θ for TE polarization. (**c**) Simulated results of the maximum reflection phase difference by varying the angle of incidence θ from 0° to 45° for TM polarization. (**d**) Reflection magnitude simulation results with varying θ for TM polarization.

The unit cell utilizing two cross elements, with full phase control, is more sensitive to angle of incidence than the previous design due to the symmetries of the periodic lattice. The maximum achievable phase difference obtained from simulations with various angles of incidence for TE and TM modes can be seen in Fig. 8a,c respectively. The reflection losses remain less than 2 dB for all angles of incidence for both orthogonal components (Fig. 8b,d), and the amount of phase shift available remains fairly stable for all but the more extreme angles exceeding 45°.

**Figure 9:**
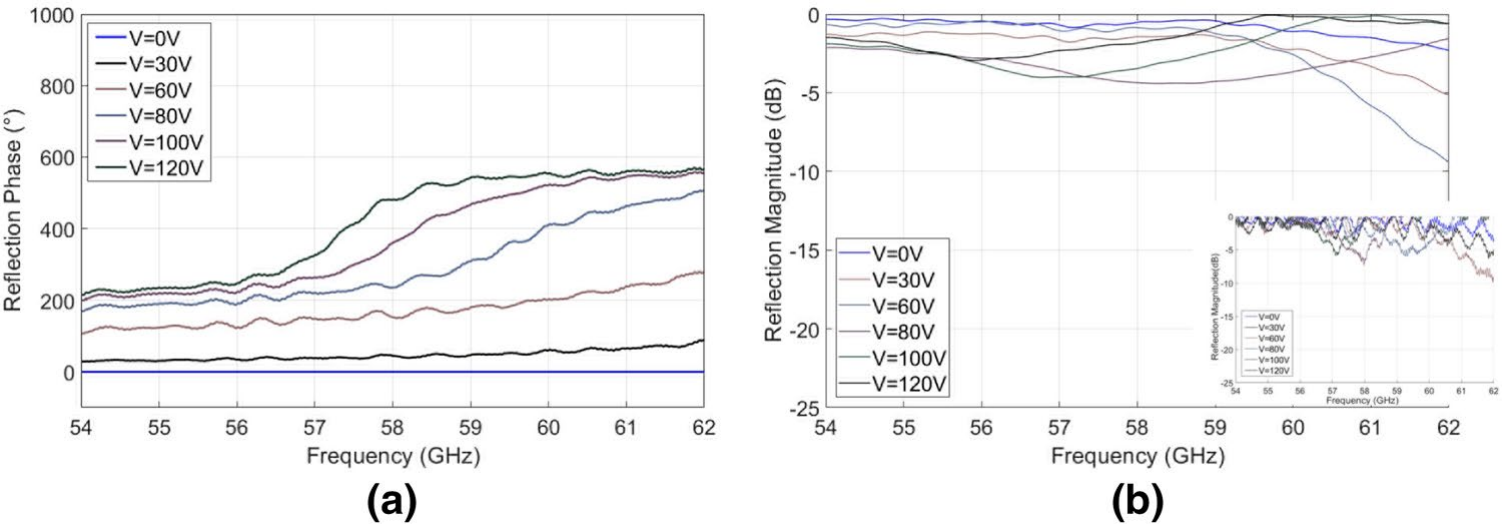
Full phase control metasurface. (**a**) Reflection phase shift with respect to 0 V (approx. 0.10 mm cavity height) state. (**b**) Average reflection magnitude for different applied voltages (raw data shown in insert).

For experimental validation, the same substrate as used previously (with thickness of 0.78 mm and dielectric constant of 2.2) was patterned with double-crosses of dimensions *p* = 2.6 mm, *w*_1_ = *w*_2_ = 0.3 mm, *l*_1_ = 0.75 mm and *l*_2_ = 0.85 mm, in a repeated array measuring 100 mm × 100 mm. The same PEA measurement technique was implemented, varying control voltage from 120 to 0 V. The results obtained can be seen in Fig. 9.

The presented results are again normalized with respect to the ground plane in the absence of the periodic surface for each measured position. Therefore, phase shifts by varying the distance from source to ground are not incorporated into these results and all phase shift is a result of the engineered impedance effects caused by the metasurface.

The maximum phase difference is 573° and is achieved at 61 GHz. The operational bandwidth where the phase shifter can achieve a greater than 360° phase shift is from 57 GHz up to 62 GHz, as expected from the simulations. The reflection magnitude is depicted in Fig. 9b, where the losses are comparable with the simulation losses, but slightly higher. This is attributed to fabrication tolerance issues along with alignment and sagging difficulties caused by the compliance of the substrate.

## Discussion

Techniques that enable the tunability of metasurfaces offer extended capabilities in the manipulation of the spectral and spatial properties of electromagnetic waves. In this report two new contributions to the state-of-the-art are presented that utilize piezo-electric tuning and extend the current possibilities of tunable metasurfaces. The loss superiority^40^ of this tuning method is enabled by the fact that the active mechanism is shielded beneath a ground plane and does not interact with the mm-wave circuit and therefore does not introduce additional losses that are inherent with other tuning technologies. This enables piezo-tuned metasurfaces to operate at mm-wave frequencies, where the design of conventional tunable devices is particularly difficult. Due to the electro-mechanical tuning nature and low loss operation, this technique is also possible at higher mm-wave bands and well into the THz band. The relative advantages of this technology, in contrast to traditional regimes, increase as frequency increases, as the amount of displacement available becomes a larger fraction of the wavelength, and thus more phase shift is possible, while maintaining losses that are as low as possible. The limits of this technology are not loss, but the accuracy of the desired movement, and as the PEA system can provide positional accuracy of about 1 µm which is sufficient to provide continuous phase shifting well into the THz region.

The two piezo-tuned structures showcased here demonstrate that with the low losses provided by this technique; a simple patch geometry can produce a 180° quasi-optical phase shifting surface with a bandwidth of 30 GHz and average losses of 1 dB, along with a full phase control phase shifting surface which can provide 5 GHz bandwidth and with average losses 1.8 dB. Both structures operate within the mm-wave band where it is traditionally difficult to achieve good loss performance.

The proposed triangular lattice double-resonance concept proposed in the second metasurface can be potentially implemented using other tuning techniques to achieve a full phase control of 360°, such as liquid crystals or reverse bias Schottky junctions, however with greater losses and less operational bandwidth.

The piezo-enabled devices used in this study pave the way for a new class of tunable metasurfaces that can be utilised in applications such as impedance tunable ground planes for printed^41^ or cavity antennas^16^, and furthermore can be used as constituent components for advanced beam-steering resonant leaky-wave antennas^42^. With modification of unit cell design, such a technique could also be used with reflectarrays^6–12^ and holographic surfaces^43^. The metasurfaces currently outlined with this new technique have used a method by which the entire periodic surface is tuned with the same amount of displacement. The next step in our research strategy is to investigate how sub-sections of the array can be tuned with varying amounts of phase shift.

In each of the proposed designs the PEA supports the ground plane and moves between around 0.10–0.50 mm from the surface of the periodic array. This is possible by the application of a control voltage on the PEA between around 24 and 120 V. The displacement of the PEA varies continuously with applied voltage, but can suffer from hysteresis effects. This was prevented in this study, by unidirectional application of voltage after initial calibration, but could be combated more effectively with the use of closed-loop feedback control that is commonly used with such devices. The PEA has inbuilt strain gauges that enable this, and commercial control circuitry is readily available.

Piezoelectric actuators have superior switching speeds when compared other mechanical movement mechanisms such as solenoids, small motors or other magnetic based devices. The flexure PEA speed is typically in the order of milliseconds and varies depending on load. For the ground plane used for the experiment a switching speed of less than two milliseconds could be expected. The combination of reliability and loss performance of piezoelectric actuation method outshines other competing technologies in this frequency range, with durability exceeding 10^9^ cycles. A potential limitation of the piezo-tuning system is the alignment tolerance, as these issues will become more problematic with increased frequency. However, with modern ultra-high precision manufacturing and metrology, and automated optical instruments, these issues should be surmountable with continued development.

## Methods

**Figure 10:**
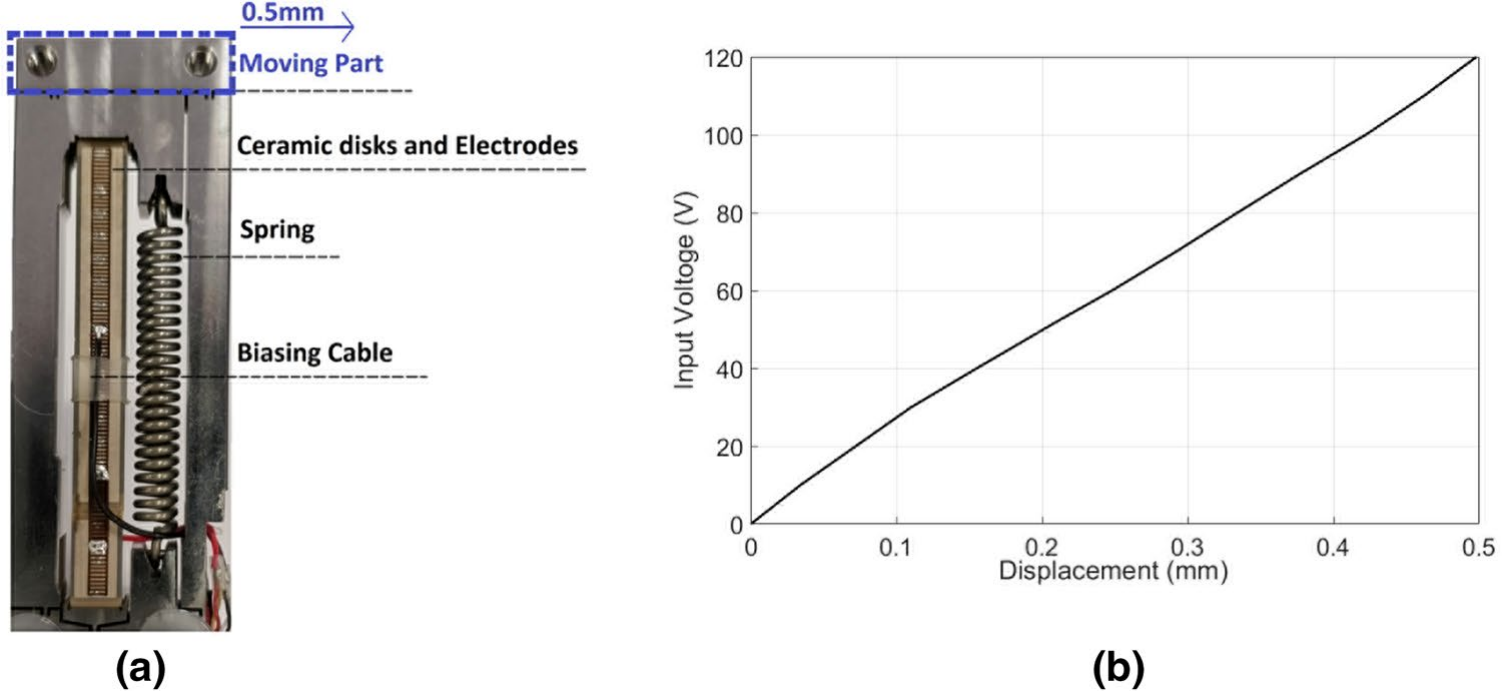
The commercial PEA used in this application. (**a**) Photograph of the PiezoMove P-603.5S1 (**b**) The displacement that provides the PiezoMove P-603.5S1 measured by microscope.

The PEA tuning technique makes use of the reverse piezoelectric effect whereby a piezoelectric material with an applied charge across its volume will produce a mechanical strain. With the commercially sourced PEAs used in these experiments, numerous thin disks of the piezoelectric material, namely lead zirconate titanate (PZT), are formed into a stack and biased via interleaved electrodes. When a biasing voltage is applied, the stack translates the resulting strain into an increase in the overall stack length. The stack is constrained within an aluminum flexure assembly which acts as a lever and amplifies the increase in length to a maximum lateral travel distance of approximately 0.50 mm. The PEA encased in the flexure assembly can be seen in Fig. 10a. The PEA used in the presented studies is the PiezoMove P-603.5S1 (by Physik Instrumente Ltd UK)^44^, and the moving part was oriented perpendicular to the ground plane and attached so to provide lateral movement.

The application of voltage between 0 and 120 V controls the position of the PEA head for a displacement between 0 and 0.50 mm (around 0.40 mm of which was used in this study). A displacement-voltage graph is shown in Fig. 10b. This graph does not show hysteresis effects, which are present, but with the unidirectional application of voltage, this graph was consistent as observed and recorded by a measurement microscope.

Each of the presented experiments was setup so that two standard gain horn antennas, of appropriate bandwidth and with minimal incidence angle, were configured to detect reflected S_21_ magnitude and phase in the far field as in Fig. 3a. Bare ground measurements were also taken with the periodic surface absent, so that free path losses may be removed, to isolate the effects of the metasurface. For the square element measurements three sets of horn antenna were required to cover the entire operational bandwidth. A moving average filter was applied to the |S_11_| data in order to reduce noise caused by standing waves which were formed as a result of the test horn antennas being placed in close proximity to one another. The close proximity of the antennas was required to minimize the angle of incidence for the reflection measurements which is desired for the intended use of such phase shifters^16,41,42^.

The two metasurface designs were first simulated and optimized for their particular objectives in an industry standard EM simulation package (CST Microwave Studio). Individual unit cells were simulated using periodic boundary conditions which yield the result of an infinite array of unit cell designs and are commonly used to model arrays sizes over 10 λ × 10 λ under plane wave incidence with very good accuracy. The PEA component was not required during simulation, as it does not form part of the EM system. Its effect was modeled by varying the air gap between periodic surface and ground plane directly.

**Figure 11:**
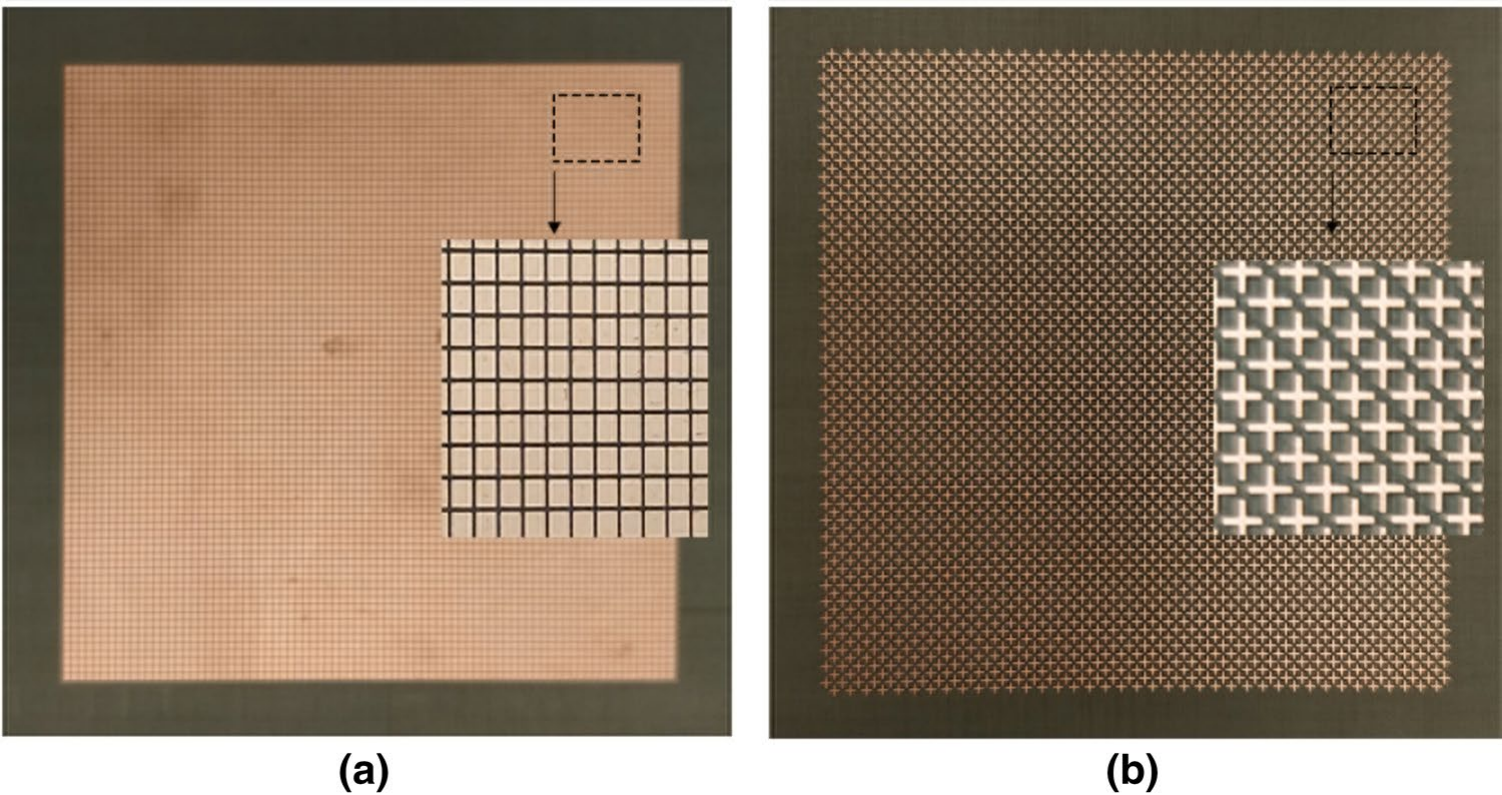
Photograph of fabricated metasurface. (**a**) Square patch element metasurface for enhanced bandwidth. (**b**) Dual cross element metasurface for full phase control.

For experimental validation standard PCB methods were sourced using a TLY-5 Taconic substrate with dielectric constant 2.2, lose tangent 0.0009 and thickness 0.78 mm. The two metasurfaces printed on the substrate had dimensions 10 λ × 10 λ at the lowest frequency which should provide a similar result to an infinite surface simulation. The wide band periodic surfaces can be seen in Fig. 11a and the full phase control variant can be seen in Fig. 11b. The periodic arrays were suspended over the variable aluminium ground plane for testing. The PEA was connected to a DC voltage source to control the displacement. By increasing the voltage, the ground plane moved away from the surface, and the air cavity height increased. A ZVA 67A vector network analyzer (Rohde & Schwarz) was used to sweep the appropriate frequency ranges. Specialized phase stable cables (ZV.796) were used to ensure accurate reporting of phase measurements.
